# MicroRNAs in oral fluids (saliva and gingival crevicular fluid) as biomarkers in orthodontics: systematic review and integrated bioinformatic analysis

**DOI:** 10.1186/s40510-021-00377-1

**Published:** 2021-10-11

**Authors:** Priyanka Kapoor, Aman Chowdhry, Dinesh Kumar Bagga, Deepak Bhargava, S. Aishwarya

**Affiliations:** 1 School of Dental Sciences, Sharda University, Greater Noida, UP India; 2 Department of Orthodontics, Faculty of Dentistry, Jamia Millia Islamia, New Delhi, 110025 India; 3 Department of Oral Pathology & Microbiology, Faculty of Dentistry, Jamia Millia Islamia, New Delhi, 110025 India; 4 Department of Orthodontics & Dentofacial Orthopaedics, School of Dental Sciences, Sharda University, Greater Noida, UP India; 5 Department of Oral Pathology & Microbiology, School of Dental Sciences, Sharda University, Greater Noida, UP India; 6 Department of Bioinformatics, Stella Maris College (Autonomous), Chennai, India

**Keywords:** MicroRNAs, Orthodontics, Biomarkers, Gingival crevicular fluid, Bioinformatics analysis, Target genes, Computational biology

## Abstract

**Background:**

MicroRNAs (miRNAs) are non-coding short, single-stranded RNA molecules that may serve as biomarkers for various inflammatory and molecular mechanisms underlying bone and tissue remodeling consequent to orthodontic force application.

**Methods:**

A thorough literature search in major databases was conducted in March 2021 to generate evidence for miRNAs in orthodontics, with prior PROSPERO registration. The initial search revealed 920 articles, subjected to strict selection criteria according to PRISMA, and resulted in final inclusion of four studies. Quality assessment by QUADAS-2 classified three studies as unclear risk-of-bias while the applicability was high. Further, bioinformatic analysis was performed to identify the target genes from the miRNA database (miRDB) and TargetScan databases and their protein-protein interaction pathways with the STRING analysis.

**Results:**

Multiple miRNAs in gingival crevicular fluid (GCF) of orthodontic patients were seen, including miRNA-21, 27(a/b), 29(a/b/c), 34,146(a/b), 101, and 214 along with matrix metalloproteinases (MMPs)-1, 2, 3, 8, 9, 14 in one study. A statistically significant increase in expression of miRNA-29a/b/c,101, 21 from pre-treatment (before initiation of retraction) was seen to reach a peak at 4–6 weeks (wk) of retraction. On the contrary, miRNA-34a showed downregulation from the 1 day to 4 wk of retraction and also, negatively correlated with MMPs-2,9,14 levels at the same observation times. The distance of canine movement showed mild correlation with miRNA-27a/b, 214 at 2 wk of retraction. Bioinformatics revealed 1213 mutual target genes which were analyzed for inter-relational pathways using Cytoscape plugin, MCODE. Further, 894 prominent protein interactions were identified from the STRING database and SMAD4, IGF1, ADAMTS6, COL4A1, COL1A1, COL3A1, FGFR1, COL19A1, FBN1, COL5A1, MGAT4A, LTBP1, MSR1, COL11A1, and COL5A3 were recognized as the hub genes. Their interactions were able to isolate multiple miRNAs: hsa-miR-34a-5p, hsa-miR-29b-2-5p, hsa-miR-29b-3p, hsa-miR-34a-3p, hsa-miR-27a-5p, hsa-miR-29a-5p, hsa-miR-29b-1-5p, hsa-miR-29c-3p, hsa-miR-214-5p, hsa-miR-27a-3p, hsa-miR-29a-3p, hsamiR-146-5p, which were found promising as biomarkers for tooth movement.

**Conclusions:**

Our results support using miRNAs as biomarkers in varied orthodontic study designs and for inter-relationships with pathological settings like periodontal disease, pre-malignancies, or conditions like obesity or metabolic irregularities, etc. The identified target genes and their protein interaction pathways can be used to propose precision therapies, focusing on ideal tooth movement with minimal iatrogenic side-effects.

**Supplementary Information:**

The online version contains supplementary material available at 10.1186/s40510-021-00377-1.

## Background

**Fig. 1:**
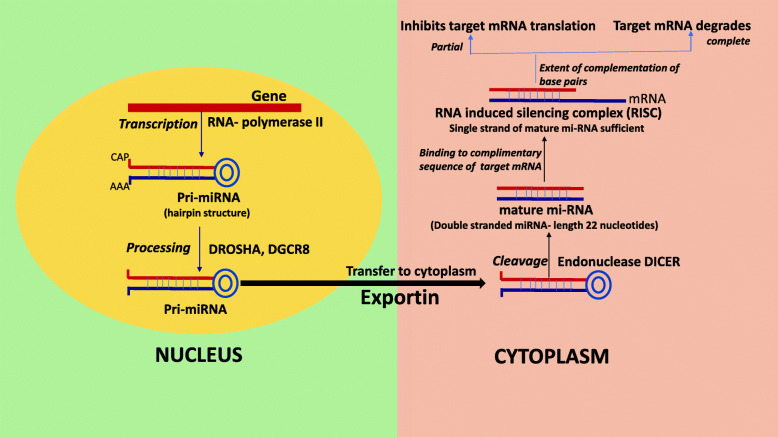
A simplified diagram explaining the pathway of microRNA (miRNA) dependent gene expression. Shows processing of three different types of miRNA, pri-miRNA in nucleus, pre-miRNA migration to cytoplasm, processing of mature mi-RNA in cytoplasm. Inputs from [1, 2]

MicroRNAs (miRNAs) are non-coding short regulatory RNAs, usually known as RNA-interfering systems or gene silencing entities, that have a significant role in various pathological and physiological activities in the body. They work by regulating gene expression by imperfect complementary binding of target messenger (mRNA) strands and hence, their derangement can easily serve as biomarkers for different disease processes or altered growth and their prognosis. The three different kinds of miRNAs, pri-miRNAs (large RNA precursors in the nucleus), pre-miRNAs (stem-loop structures transported into the cytoplasm), or mature-miRNAs and their mechanism of action has been depicted diagrammatically in Fig. 1 [1, 2]. miRNAs can singularly affect multiple strands of mRNAs, alter the gene expression profile of target genes and their pathways, hence can influence multiple cell-regulating functions by altering the protein–protein interactions, ultimately affecting causation of disease and its severity [3, 4].

### MiRNAs in dentistry

Literature evidence suggests an association of miRNAs, specifically miR-34a with the development of the tooth, remodeling of bone, as well as differentiation of dental stem cells [5–7]. Besides, various miRNAs are associated with oral cancers as well as with a higher risk of conversion of oral premalignant lesions to malignancy. Change in expression of several miRNAs has been documented with related risk factors for oral cancer, e.g., increased miR-31 and miR-138 expression and decreased miR-10b, miR-92a, miR-200b, miR-372, miR-375, miR-378a, and miR-145 expression with smokeless tobacco use. Further, research is being conducted to explore deregulated miRNAs as biomarkers for clinic-pathological indicators, precision treatment, and generation of expression profiles for oral cancer [8].

### miRNAs in craniofacial aberrations

miRNAs expression and function have also been associated with the development of the face, specifically secondary palate growth [9, 10]. A recent systematic review by Mendes et al. 2020 has reported upregulation of 57 and downregulation of 43 out of 100 mi-RNAs in different categories of cleft lip and palate (CLP) and additionally, differential expression of 9 mi-RNAs in cleft palate (CP) patients [11]. A single study on salivary miRNAs in CLP patients by Grassia et al. (2017) has revealed miR-324-3, p miR-141, and miR223 as probable biomarkers of CLP  malformation [3].

### miRNAs in orthodontics

Attempts have been made to study miRNAs, both in mice and humans, upon application of orthodontic forces as mechanical stimuli are known to initiate a cascade of cellular and biochemical processes after the release of various pro-inflammatory markers which mediate the remodeling of bone and periodontium. These mediators are known to play an active role in osteoclastogenesis [e.g., interleukin (IL-1β), tumor necrosis factor (TNF)-α, receptor activator of nuclear kappa ligand (RANKL), etc.] as well as osteoblastogenesis (e.g., osteoprotegrin (OPG), IL-4, IL-10, etc.] [12, 13]. Additionally, the expression of different miRNAs at multiple observation times in orthodontic tooth movement (OTM) are correlated with osteoclastic/osteoblastic differentiation associated with the release of multiple biomarkers in the paracrine environment [7, 14, 15].

### miRNA detection

Secretory miRNA can be detected in body fluids like the whole serum, urine, and cerebrospinal fluid as well as in oral fluids like saliva and gingival crevicular fluid (GCF). Their presence is usually detected in exosomes, which are micro-vesicles, very small (30–100 nm) in size, and carry various nucleic acids and proteins [16]. It is well documented by utilizing quantitative polymerase chain reaction (qPCR) that miRNA in exosomes in saliva/serum, when compared to exosome-depleted serum/saliva and whole serum, are much more in number and can be detected even in very small quantities [14]. Besides, in orthodontic treatment, oral biofluids including GCF and saliva may be the most convenient and patient-compliant source of identifying these miRNA biomarkers.

### Bioinformatics in orthodontics

Bioinformatics involves biological abstracting in the context of macromolecules and application of principles of “informatics” (using computational domains: applied maths, statistics, and computer science) to organize the evidence gathered from these molecules and comprehend the derivations on a larger scale for application in multiple fields of biology [17]. The advent of bioinformatics has shown tremendous potential to bridge the gap between basic research and medical or dental applied sciences. Clinical orthodontics entails a complex phenomenon of hard tissue and soft tissue remodeling involving complex interaction of a multitude of cells consequent to force application, which is difficult to summarize and interpret. Janjic et al. (2018) have utilized bioinformatics to investigate stress adaptation of cells upon application of different types of in vitro mechanical loading models in a systematic review and used the STRING analysis to outline the most significant protein-protein interaction (PPI) networks and signaling pathways [18]. Another in vitro study used bioinformatic analysis to screen the candidate genes and pathways in response to mechanical force on osteocytes, to derive that hypoxia might be the key factor of differential response [19]. A recent in vivo study in mice by Klein Y et al. (2020) identified differential gene expression (DEG) upon coil spring activation at different time points and presented a heat map of “top 50” genes showing significant changes, along with two distinct DEG clusters: one showing initial downregulation in 3 days (d), followed by upregulation (representing formative events of cellular proliferation, migration, etc.) until 14 d while the other peaked initially, only to decrease in 14 d (representing inflammatory degradative, innate/adaptive immune responses, etc.) [4]. Continuing research in this domain has been contemplated to merge genetics, biology, and orthodontic therapeutics to formulate the basis for precision orthodontics and a personalized treatment approach [20].

### Gap in literature

Although various biomarkers have been identified in oral fluids at the biochemical level, the evidence is still sparse related to post-transcriptional regulation of the associated changes by the miRNAs. Hence, a critical evaluation of existing literature is required to identify the most potent miRNA biomarkers in OTM, their time-dependent alteration, and their effect on the gene expression profile and protein-protein interactions (PPI), to understand the biology of hard and soft tissue alterations and remodeling.

Additionally, target genes of the identified functional miRNAs were corroborated, and a regulatory co-expression network of the target genes and miRNAs was constructed to analyze the underlying protein interactions. This will provide a basis for understanding specific biological collaborations in tooth movement and may further the scope of precision therapies and research opportunities in this domain. Further, the protein interactions causing iatrogenic side-effects like apical root resorption or caries-risk can also be identified.

## Aim and objectives

The current study was conducted with the following objectives:

1. To study expression of miRNAs in oral fluids (GCF and saliva) of patients undergoing orthodontic treatment.
2. To identify the target genes of the miRNAs isolated in orthodontic patients by using miRNA database (miRDB) and TargetScan databases.
3. To analyze protein-protein interactions and identify HUB genes using Cytoscape plugin, Molecular Complex Detection (MCODE).

## Material and methods

A critical review of the literature was performed to explore the association of miRNA in oral fluids orthodontics, after registration of protocol in PROSPERO (CRD42021244200). A thorough literature search was conducted in major databases—PubMed (P), Web of Science (WOS), J-Gate, Directory of Open access journals (DOAJ), Scopus (S), Embase, open access thesis and dissertations (OATD), hand search (HS), and reference tracking in March 2021. There was no limitation of date or language in search. Both MeSH and free text terms were used for the search: “microRNA,” “miRNA,” and “Orthodontics.”

The following search strategy was used:

PubMed/ web of Science/Scopus: (microRNAs) OR (miRNA) AND (Orthodontic*)

Embase: (“microrna”/exp OR microrna) AND (“mirna”/exp OR mirna) AND (“orthodontics”/exp OR orthodontics)

DOAJ/ OATD/J-Gate: (microRNAs) OR (miRNA) AND (Orthodontics)

**Fig. 2:**
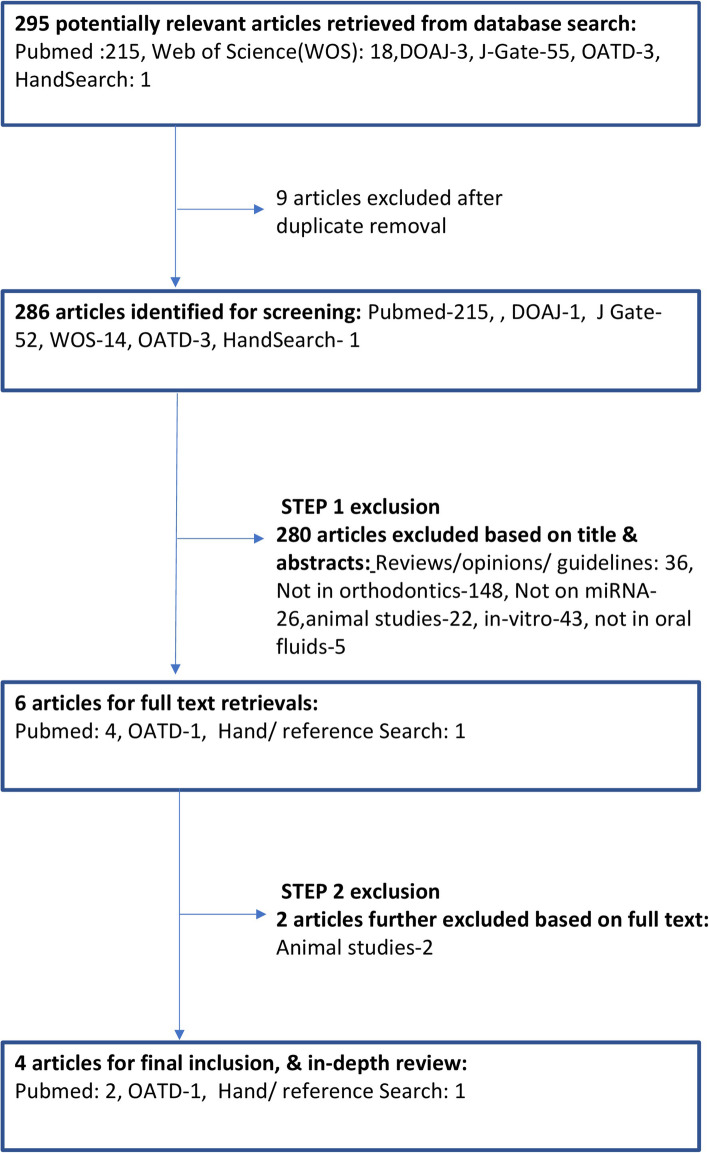
Details of the preferred reporting items of systematic reviews and meta-analysis (PRISMA)

The initial search was applied by two researchers independently (PK and AC). It revealed 920 articles including 215 in (P), 364 in (S), 261 in Embase, 18 in WOS, 55 in J-Gate, 3 in DOAJ, 3 in OATD, and 1 in (HS) [details of the preferred reporting items of systematic reviews and meta-analysis (PRISMA) given in Fig. 2]. After duplicates removal, strict inclusion and exclusion criteria (Table 1) were applied which yielded six articles [4 in (P), 1 in OATD, 1 in (HS)]. Further exclusion of 2 articles was done due to studies on animal experiments . This led to shortlisting of 4 studies [2 in (P), 1 in (HS), 1 in OATD] which were subjected to subsequent quality assessment. The data extraction of the final studies was performed by two investigators (PK, AC) independently based on participant characteristics, study design, miRNA studied, oral fluid for isolation, and outcomes (Table 2). Any discordance in any of the data was discussed with a third researcher (DKB) and resolution was done after reaching a consensus.

**Table 1:** Inclusion and Exclusion criteria for studies

Inclusion	Exclusion
All original studies on humans including clinical trials, prospective or retrospective cohort studies, studies mentioning both miRNA and tooth movement	In-vitro studies, animal studies, studies on miRNAs but not in orthodontics, studies in orthodontics but not in miRNAs, miRNAs not measured in oral fluids, case reports, and reviews/opinions/guidelines

**Table 2:** Presentation of evidence related to microRNAs in orthodontics based on participants and study characteristics, GCF handling, and outcomes

S No.	Authors	Age/sex of study participants	Oral fluid	miRNA studied	Observation times	Study characteristics	GCF Collection	Handling	Analysis	Results	Conclusion
1	[21]	15 (10–17 y) patients for test sample, 4M (26–28 y) for std. sample	GCF	miRNA-29 (a/b/c)	5 times, T0-prior to retr., T1-1 hr post retr., T2-1 d post retr., T3-7 d post retr., T4-6 wk post retr.	CaninerRetr. force of 250 g	Periopaper, Mesial site of canine	1min. placement, repeat 4 times	Quan. & qual. of RNA; Bioanalyzer, spectrophotometer, secretory miRNA presence: miScript PCR array, miRNA-29 presence: RT-real-ime PCR	Stat. sign. increase in levels of miRNA-29 family from T0-T4,-specificall y in miRNA-29b: from T0-T1, T0-T3, no stat. diff b/w levels of miRNA 29-a,b,c	Presence of miRNA-29 in GCF (mostly in exosome) in OTM, role in regulation of osteoclasts & ECM molecule expression
2	[7]	20 (12–18 y)	GCF	miR-34a and MMPs-1, 2, 3, 8, 9, 14	6 times, T0-day of force application, T1-1 hr post retr., T2-1 d post retr., T3-1 wk post retr., T4-4 wk post retr., T5-12 wk post retr.	Canine retr. as explained in [48], oral hygiene protocol of 0.12% CLX gluconate rinse for 1 wk, random canine as index tooth, contra. canine as control, canine retr.	Periopaper, tension & compr. sites	1min. placement	RT-PCR	T2–T4: mi-34a decrease & MMPs increase compared to T0, T1, T5: baseline levels, -ve correl. of miR-34a with MMP-2, 9, 14, no diff. b/w tension & compr. sites, no variation in control teeth	miRNA-34a role in OTM, regulates osteogenic function, Wnt/b-catenin pathway
3	[2]	11 (3M, 8F, 10-18y), ethnic distr.: 9 Hispanics, 1 African American, 1 Caucasian.	GCF	miRNA 29 (a,b,c), 21, 101	6 times, T0-before bonding, T1-0 d of canine retr. (before retr.), T2-1 hr post retr., T3-1 d post retr., T4-7 d post retr., T5- 5 wk post retr.	Canine retr. 250 g, by E-chain activn	Periopaper, MB DB sites	1–2 mm insertion, 4 times collection (60 sec each)	Total RNA: spectrophotometer, specific miRNA: qRT-PCR, RNA assay, real-time PCR system	miRNA 29a: increase b/w T1–T2, fall in T3, peak at T5 (stat. sign.) miRNA 29b; stat. sign increase b/w T1–T2, T1–T4, T1–T5, miRNA 29c stat. sign. increase b/w T1–T5, miRNA 101: stat. sign. increase b/w T1–T4, T1–T5, miRNA 21: stat. sign. increase b/w T1-T5, no stat. diff. b/w levels of 29a,b,c	miRNAs-29a/b/c, miRNA-21 role in osteoclast & osteoblast regulation, miRNA 101:against fibrogenesis
4	[22]	11 (4M, 7F, 14.75 y av. age))	GCF	miRNAs 27 (a/b), 146(a/b), 214	5 times, T0-prior to bonding, T1-0 d (before coil spring activn.), T2-2 wk post retr., T3-5 wk post retr., T4-7 wk post retr.	Canine retr. with 150cN closed coil spring anchored to sectional L-loop &TADs	Periopaper	5 periopaper strips at each time point (60 sec each), 1^st^ for volumetric & rest 4 for miRNA measurements	Real-time qRT-PCR, spectrophotometer	Canine m/ment distance b/w T1-T2 showed stat. sign. +ve correl. with profile express. of miRNA-27a at T2, miRNA27b at T2, miRNA 214 at T2, b/w T1-T3 with profile express. of miRNA 27b at T3, no change in miRNA 146a/b, mild -ve correl. b/w GCF volume & miRNA-27a change at T4	Role of miRNA 27a/b, 146a/b, 214 in OTM, of which expression profile of miRN27a/b, 214 showed correl with canine m/ment, role in catbolic & anabolic pathways

+*ve* positive, −*ve* negative, *activn* activation, *b*/*w* between, *CLX* Chlorhexidine, *compr*. compression, *correl*. correlation, *d* day(s), *DB* distobuccal, *diff*. difference, *distr*. distribution, *E*-*chain* elastic chain, *ECM* extracellular matrix, *hr* hour(s), *GCF* gingival crevicular fluid, *max*. maximum, *MB* mesiobuccal, *min*.(s) minutes, *miR*/*miRNA* microRNA (mature form), *m*/*ment* movement, *MMP* matrix metalloproteinases, *mon* months, *NiTi* nickel titanium, *OPG* osteoprotegrin, *OTM* orthodontic tooth movement, *PCR* polymerase chain reaction, *pdl* periodontal ligament, *Qual*. quality, *Quan*. quantity, *RANKL* receptor activator of n-Kappa ligand, *retr*. retraction, *sec*. second(s), *sign*. significant, *stat*. statistically, *std*. standard, *TAD* temporary anchorage devices (TADs), *wk* week(s), *WT* wild type, *y* year(s)

## Quality assessment

The QUADAS (Quality Assessment of Diagnostic Accuracy Studies)-2 tool was used to evaluate risk of bias (ROB) for each study [23]. It was performed independently by two researchers and in discrepancy, consensus by referring to a third evaluator (an additional file shows this in more detail [see Additional file 1]). Three out of four studies had an unclear ROB while applicability was high.

### Target gene prediction

Further, the target genes for the identified functional miRNAs were analyzed by TargetScan which predicts the targets by computing the 8mer, 7mer, and 6mer binding sites of miRNAs [24], as well as MicroRNA Target Prediction Database (miRDB) which identifies the targets from the high throughput sequencing experiments and machine learning methods [25]. To enhance the credibility and reliability of target prediction, mutual genes were selected from both miRDB and TargetScan databases.

### Gene annotation and pathway analysis

Annotation of the target genes offers significant meaning to the genes. Inter-relational gene annotation was performed with the ClueGO application (version 2.5.7) [26] installed in the Cytoscape software (version 3.5.10). Benjamini Hochberg statistical validation with a kappa score of 3, *p* value < 0.05 annotated the genes for their biological process, molecular function, cellular components, Kyoto Encyclopedia of Genes and Genomes (KEGG) pathways, and immune reactions [27].

### Protein interaction network and hub genes prediction

Protein-protein interaction (PPI) and co-expression networks for the encoding of mutual target genes were evaluated using the STRING database [28]. The potential interactions were filtered out with the MCODE application installed in the Cytoscape software. Interacting proteins and the co-expressing proteins were clustered based on the haircut option with the networks scoring degree cutoff of 2, K-score of 2, and node score cutoff of 0.2. The cluster with the highest cluster score was selected as the hub genes [27].

## Results

The results have been presented in the following sequence:

I. Dynamics of miRNAs in oral fluids
II. Target gene identification and annotation
III. Regulatory co-expression network and hub gene prediction

### Dynamics of miRNAs in oral fluids

The results of the studies are tabulated and presented in Table 2, divided into the following categories:

#### Participant characteristics

The sample size in the included studies was 11 in two studies [2, 22], *n* = 15 and *n* = 20 in one study each. Studies also specify dropouts, as well as the exclusion of participants from the study due to gingival conditions [220] [2, 22]. Male to female ratio has not been mentioned in two studies [7, 21]. Ethnic distribution of participants was mentioned in one study, although the sample size was small [2].

#### Study characteristics

All four studies were longitudinal where sample collection was done at multiple observation points [2, 7, 21, 22]. The baseline levels of all miRNAs served as internal controls. One study also mentioned contralateral canine as control [7]. All four studies have evaluated a protocol of canine retraction with the extraction of first premolars. Observation times ranged from five times [21, 22] to six times in two studies [2, 7] over 5–6 weeks (wk) after initiation of retraction. Observation times: before initiation of retraction, immediately after retraction start, 1 day (d), 7 days were considered in all the studies. All studies analyzed miRNAs in gingival crevicular fluid (GCF) and none of the studies analyzed saliva.

#### Collection of GCF

GCF collection was done with a periopaper in all four studies [2, 7, 21, 22], with the depth of insertion of paper mentioned in one study [2]. The time of placement of 1 min (min.) was specified in all four studies but repeated placement of up to 4 times as specified in one study [2]. Site-specific collection of GCF was done in three out of four studies with the mesial site of canine in one study [21] and both the mesial and distal sites mentioned in two studies [2, 7] of which one study analyzed the difference in miRNA level between tension and compression sites [2].

#### miRNA studied

Different miRNAs have been evaluated in all the studies: miRNA-29 in two studies [214] [2, 21], miR-34a along with inflammatory mediators-MMPs [7], miRNA-21 [2], 101 [2], 27 [22], 146 [22], and 214 [22] in one study each.

#### Upregulation or downregulation of miRNAs

Expression profile of miRNA varied in GCF at different observation times. There was a statistically significant increase in levels of miRNA-29 family from pre-retraction levels to 5–6 weeks post-retraction, with no difference in levels of miRNA 29a/b/c [214] [2, 21]. Specific components of miRNA-29 family showed differential expression with the application of retraction forces: miRNA-29a increases from baseline to 1 h (h) post-retraction followed by a decrease at 1day and further peak at 5 weeks [2]. miRNA-29b showed a statistically significant increase from pre-retraction to 1 h and 7 days [214] [2, 21]. miRNA-29c showed a significant increase from 1 h post- retraction to peak at 5 weeks [2]. Besides, miRNA-34a showed a significant decrease from 1 day to 4 weeks post retraction compared to baseline and 1 h post-retraction while a negative correlation was seen in matrix metalloproteinases (MMPs)-2,9,14 with an increase in levels in the same time intervals, both on tension and compression sites [7]. miRNA-101 showed a statistically significant increase from 1 h post-retraction to 7 days and 5 weeks while miRNA-21 showed a significant increase at 5-weeks time interval [2]. Family of miRNA-27, 214, 146 showed a variation related to the distance of canine movement during retraction: levels of miRNA-27a, miRNA-27b, and miRNA-214 showed a positive correlation with the canine distance in these 2 weeks. miRNA-27b levels also showed a positive correlation of canine distance at 5 weeks of retraction but no significant correlation was observed in miRNA-146(a/b) [22].

### Target gene identification and annotation

Once the miRNAs were identified in the oral biofluids, they were studied for the target genes by Bioinformatics software. The target genes were retrieved from established miRNA databases by a Bioinformatician (AS), further subjected to identification of mutual genes by scoring above an established cut-off value. To read these target genes, it is important to understand a few terminologies. The component “hsa” refers to human miRNA, the number 27, 29 etc., refers to the stage at which the miRNA was discovered: the lesser the number, the earlier it was discovered. The term 5p or 3p in the precursor miRNA stem loop structure means either it is from the 5 prime arm of the hairpin or from 3 prime end respectively. After the identification of target genes, the detailed pathways of action were identified which may play a role in orthodontic remodeling of bone, collagen, extra-cellular matrix, and many signaling pathways.

#### Target gene identification

**Fig. 3:**
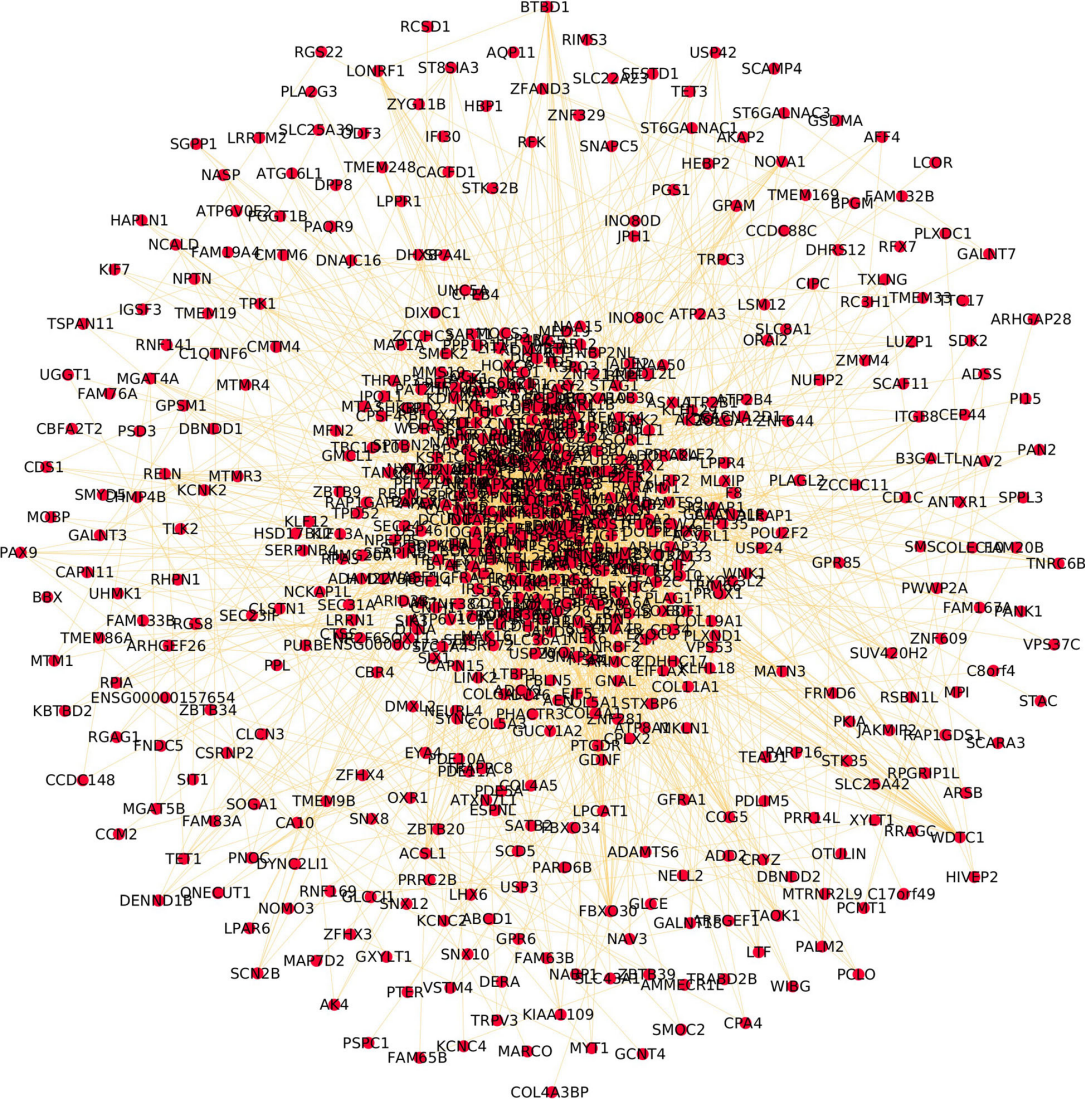
Results of target gene identification from miRDB and TargetScan databases

The target genes for hsa-miR-27a-3p, hsa-miR-27a-5p, hsa-miR-27b-3p, hsa-miR-27b-5p, hsa-miR-29a-3p, hsa-miR-29a-5p, hsa-miR-29b-1-5p, hsa-miR-29b-2-5p, hsa-miR-29b-3p, hsa-miR-29c-3p, hsa-miR-29c-5p, hsa-miR-34a-3p, hsa-miR-34a-5p, hsa-miR-214-3p, hsa-miR-214-5p, hsa-miR-146a-3p, and hsa-miR-146a-5p as identified from the review were predicted by both TargetScan and miRDB databases. The genes were retrieved from miRDB with the score > 90 and from TargetScan ranked by cumulative weighted context++ score >− 80. There were 1213 mutual genes from both the databases that were analyzed further (Fig. 3).

#### Target gene annotation

**Fig. 4:**
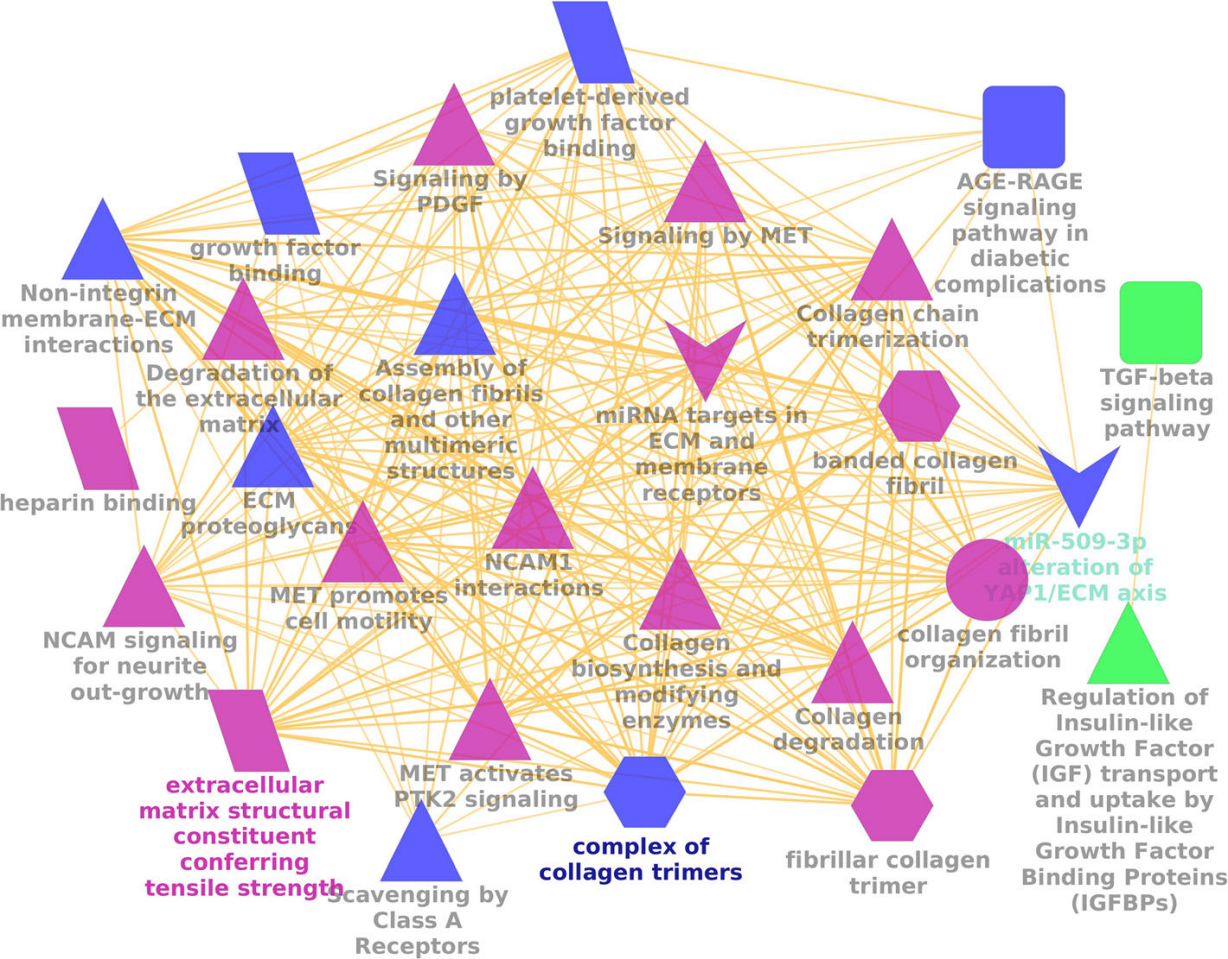
Inter-relational pathway analysis and GO enrichment of the target gene clusters. The pathways are shown in arrow and triangle representation and the GO in ellipses (biological process), hexagon (molecular function), and parallelogram (cellular components)

Inter-relational gene annotation resulted in detailing and pathways of the mutual target genes. *MCODE*, a Cytoscape plugin, found out the most significant clusters of protein interactions (highly interconnected regions) with the highest score of 68.37, with 40 gene ontology (GO)-enriched terms with 100% confidence. Figure 4 represents the complex of collagen trimers, extracellular matrix (ECM) structural constituent conferring tensile strength, collagen degradation, collagen biosynthesis, ECM degradation were significant GO terms annotated for the cluster. The target genes suppressed by the miRNAs were also responsible in insulin growth factor (IGF-1) binding, transforming growth factor (TGF)-β signaling pathways, scavenging class A receptors, and many more.

### Regulatory co-expression network and Hub gene prediction

**Fig. 5:**
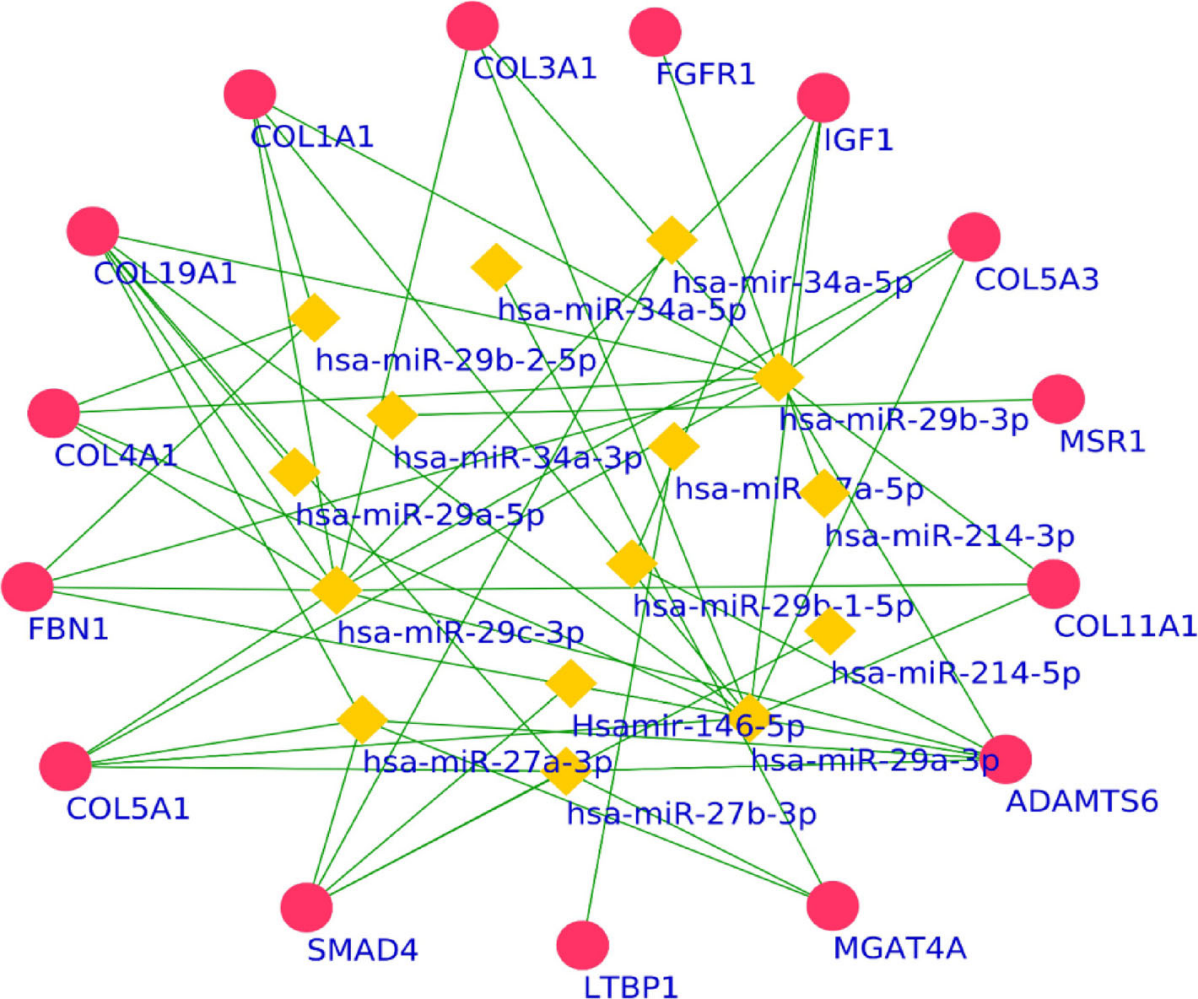
Cluster of target genes (pink circle) with the microRNAs (yellow rhombus) and potential hub genes

Protein-protein interactions (PPI) encoded by the significant targets identified from the STRING database showed 894 prominent interactions. These interactions were used to identify the Hub genes, which are described as genes with elevated association in candidate modules, which in this case are the protein -protein interactions. The score depicting the high connectivity gives an indication of the significance of HUB genes. In the current review, the hub genes as identified from MCODE resulted in a prominent cluster with the highest score of 8.98 with 15 nodes (hub genes) and 34 degrees (interactions). The hub genes were identified as SMAD4, IGF1, ADAMTS6, COL4A1, COL1A1, COL3A1, FGFR1, COL19A1, FBN1, COL5A1, MGAT4A, LTBP1, MSR1, COL11A1, and COL5A3. The interactions of hub genes along with the promising miRNAs are shown in Fig. 4. The promising miRNAs were hsa-miR-34a-5p, hsa-miR-29b-2-5p, hsa-miR-29b-3p, hsa-miR-34a-3p, hsa-miR-27a-5p, hsa-miR-29a-5p, hsa-miR-29b-1-5p, hsa-miR-29c-3p, hsa-miR-214-5p, hsa-miR-27a-3p, hsa-miR-29a-3p, and hsamiR-146-5p (Fig. 5). These hub genes can further be studied for their individualized effects on orthodontic treatment.

## Discussion

### miRNAs as biomarkers

MicroRNAs (miRNAs) are small (22 nucleotides length approximately) single-stranded, noncoding RNAs that regulate distinct biological processes by post-translational regression or destruction of mRNA target genes. They have been explored previously as biomarkers in multiple pathologic conditions like neoplasms, malignancies, transplant rejection, infection, cardiac injury, etc. [29]. Specifically, in dentistry, their role has been established as prognostic and diagnostic markers in OSCC (oral squamous cell carcinoma) [30], premalignant conditions, e.g., dysplasia in leukoplakia and conversion to malignancy [31], periodontal disease and homeostasis [29, 32], and also in craniofacial malformations including CLP [3]. Nevertheless, the role of miRNAs in OTM and remodeling has not been explored sufficiently to date [29].

### Scope for miRNA markers in OTM

The orthodontic force application is known to initiate an aseptic inflammatory cascade mediated by underlying cellular and molecular pathways. At cellular levels, the initial catabolic phase of destruction with osteoclast recruitment is supported by the release of various pro-inflammatory cytokines from pdl fibroblasts followed by leucocyte extravasation. This is followed by an anabolic phase of osteoblastic differentiation for restoration of bone and tissue architecture. However, the precise time-dependent sequence of appearance of biological mediators and processes, are still unclear. Hence, critical appraisal of the evidence associated with the presence and alteration in levels of miRNAs in human OTM studies has been attempted in the current review, to give a precise indication of differentially expressed gene (DEG) targets (given in Fig. 4), which have further indicated the pathways of gene clusters and protein interactions (given in Fig. 5) and can be explored further for different force levels and time points. The knowledge can form a strong foundation for future research into OTM biology and remodeling, furthering the concepts of precision therapies for acceleration and tooth movement without any iatrogenic side effects. Although a recent study by Klein et al. (2020) has attempted to study differential miRNA expression and associated gene targets in mice upon activation of coil spring at different time-points, their immuno-orthodontics may not be a replica of human OTM and the similarity of DEGs require validation in humans [4]. Nevertheless, this study might form a basis for comparison with the current evidence of our systematic review on miRNAs in human OTM and may strengthen the integrated bioinformatic analysis for hub gene prediction.

### miRNAs in bio-fluids

The majority of miRNAs are found in large quantities in serum and plasma, but now they are being explored in other biofluids like GCF and saliva. The studies included in the current review have all been performed in GCF as it is present near the orthodontic force application. The miRNAs are usually associated with the extracellular vesicle component in bio-fluids, as seen in the current review, and have huge potential for marker miRNA isolation as well as target gene therapy [33].

Although salivary miRNAs have not yet been explored in OTM, they are being sufficiently investigated in early screening of pathological conditions like chronic periodontitis, OSCC, Sjorgen syndrome, etc., and have also been established in developmental defects like CLP [3, 34, 35]. Due to their ease of collection, cost-effectiveness, repeated sampling, and use in large population based, salivary biomarkers are fast developing as point-of-care diagnostics, even for the current COVID-19 pandemic [29, 36]. Hence, the current review supports future research in the salivary miRNA domain in orthodontics which can also be studied for target genes for overlapping gingival and periodontal conditions which might influence tooth movement.

### Epigenetic regulation of miRNA in OTM and target gene identification

The present review identified multiple families of mi-RNAs in humans during orthodontic canine retraction, including miRNAs-21, 27, 29, 34,146, 214, 101. Each of them is associated with varied molecular mechanisms playing a role in the remodeling of bone and surrounding structures in tooth movement.

Of these, miRNA-21, which is one of the earliest discovered miRNAs, show a steady rise in miRNA-21 from 1^st^–5^th^ week of application of 250 g of retraction force in a study by Lazari P. 2016, included in our review [2]. The role of miRNA-21 has been studied in tooth movement in mice which indicates that the post-transcription control of protein mediated by miRNA-21 leads to inhibition of target gene PDCD4, which in turn regulates C-Fos, further potentiating osteoclastogenesis [37]. Thus, the phase of the tooth movement which supports rise in miRNA-21 leads to heightened resorptive activity. This finding is supported by Zhang et al. (2020) in a study on 36 male Sprague-Dawley rats which evaluated miR-21and its target genes in periodontally accelerated osteogenic orthodontics (PAOO) with an orthodontic loading of 25 g by a tension spring between central incisors and maxillary first molar. Results showed a rise in miRNA-21 levels in combined PAOO and tooth movement compared to the latter alone, after 7 days of surgery [37]. Further, this study also reported higher miRNA-21 associated with down streaming of target mRNA and PDCD4 protein expression leading to increased expression of RANKL and C-Fos proteins. Similar target genes modulating clastogenesis like RANKL have been identified in TargetScan and miRDB databases in the current review and also in in-vivo gene expression in mice in a study by Klein et al. 2020 [4]. Another study by Cheng et al. (2016) used 30 g force to evaluate miRNA-21 expression in 3-month-old WT (wild type) and miR-21^−/−^ mice and showed that OTM distance in miR-21^−/−^ mice was 30% that of WT mice and also, a significantly higher miR-21 was observed at 7 days of OTM compared with baseline levels, thus supporting role of miRNA-21 in tooth movement, as seen in the current review [38]. Additionally, Wu et al. (2020) also showed retarded tooth movement in miR-21^−/−^ mice due to decreased osteoclast numbers and suppressed RANKL pathway, thus supporting the target gene for resorption identified in the bioinformatic analysis in the current review [15]. The role of miRNA-21 in osteoclastogenesis has also been established by in-vitro studies, one by Kagiya and Nakamura (2013) on cells treated with TNF-α/RANKL, which showed increased expression of miRNA-21 and in-turn differentiation of osteoclasts while another study by Sugatani et al. (2011) showed that miRNA-21 silencing downregulated osteoclastogenesis [39, 40].

Another family of miRNA studied in the present review was miRNA-29 and its role in orthodontic tooth retraction evaluated in two studies. While one study in the current review showed a statistically significant peak in the levels of miRNA-29a/b/c at 5 weeks of retraction compared to baseline (before retraction) [2], another study showed an immediate increase in secretory miRNA-29(a/b/c), followed by leveling at 1 h and a gradual increase till 6 weeks post retraction [2, 14]. This expression profile of miRNA-29 has been previously associated with osteoclast differentiation, TRAP^+^ cell formation, RANKL production, multinucleated osteoclast formation, and extracellular matrix trabecular synthesis [39, 41, 42]. One previous study by Franceschetti et al. (2013) demonstrated the target genes influenced by miRNA-29 to better comprehend their role in osteoclast regulation. They identified negative regulation of *Srgap2* (SLIT-ROBO Rho GTPase-activating protein 2) and *Cdc42* (cell division control protein 42) in the cytoskeletal organization as well as targets like *Gpr85* (G protein-coupled receptor 85), *Nfia* (nuclear factor I/A), *Cd93* of macrophage lineage*,* and *Calcr* (calcitonin receptor) for osteoclast survival [41] Another aspect of periodontal remodeling in OTM, which is extracellular matrix (ECM) gene regulation, was also studied by Chen et al. (2015) by subjecting periodontal ligament (pdl) cells to cyclic stretch and compression forces and study interactions of miR-29b mimic/inhibitor and collagen regulator genes (COL1A1, COL3A1, COL5A1) genes, thus giving evidence of its role in ECM regulation [43]. The same target genes have been identified in the overlap of TargetScan and miRDB databases in the current review and the pathway of their action has been highlighted in Fig. 4. Contrastingly, miR-29 is also known to be involved in the anabolic process leading to differentiation of osteoblasts and downregulation of RANKL by the Wnt pathway [44, 45]. A study by Li et al. (2009) provides evidence of miRNA-29b reaching a peak at 28 d during the deposition period and causing ECM accumulation and targeting inhibitors of osteoblastogenesis [45] Similar results were seen by Kapinas et al. (2009) who additionally provided the gene targets of miRNA-29b by downregulating inhibitors of osteoblastogenesis as well as showed increased miRNA-29a/c during osteoblastic differentiation [44]. This may well explain the late peak in miRNA-29 at 5–6 wk of retraction in the current review, which corresponds to the depository phase in the studies included in our review.

Another marker miRNA, miRNA-101, showed a statistically significant increase at 1 wk and 5 wk of retraction from the pre-treatment levels in a study by Lazari et al. (2016) in the current review [2]. This increase in expression is supported by a previous study by Li et al. (2012) which identified the proteins modulated by miRNA-101- periodontal ligament-associated protein-1 (PLAP-1) as well as transforming growth factor (TGF-β), which are both responsible for anabolic processes, thus explaining the late increase in levels of miRNA-101 [46]. TGF-β signaling has also been indicated in the gene targets identified in the bioinformatic analysis of the current review, highlighted in Fig. 3. Additionally, the bone apposition process has also been linked to the presence of miR-34a, which is known for osteogenesis and angiogenesis [47].

The current review also shows miR-34a downregulation in orthodontic canine retraction from 1 d to 1 month (mon) post-retraction, both on tension and compression sites and in a negative correlation of MMPs-2,9,14 expression at the same observation times [7], whereby MMPs are known for matrix degradation [13].

MiRNA-27 family, studied in the current review, is known to suppress the pro-inflammatory response consequent to the force application. MiRNA-27(a/b) supports an increase in differentiation of osteoblasts, enabling the Wnt pathway, hence the anabolic process but the change is not statistically significant, probably due to sampling limitations [22]. Distance moved by the canine in 2 weeks and 5 weeks has shown a mild positive correlation with miRNA-27(a/b) which may correspond to the peak in osteoid mineralization and differentiation of osteoblasts [48].

An action similar to miRNA-27 is performed by miRNA-146(a/b) which is elevated in acute inflammation of OTM but when inflammation subsides, only miRNA-146a was shown to return to baseline in the current review [22], as has been witnessed in previous studies [49]. The role of miR-146 in the inflammatory cascade is potentiated by suppressing the pro-inflammatory mitogen-activated protein kinase (MAPK) and NF-κB pathway, regressing the early growth response and endothelial activation, thus serving as negative feedback mechanisms for the inflammation [49].

Besides, another unpublished thesis by Christyne Chmil (May 2020) in support of the current review, also showed an evidence of significant change in miRNA-155 expression in GCF in 2 weeks of retraction by micro-implant supported retraction using closed coil spring (*p* value = 0.006). Other miRNAs, miRNA-21, and miRNA-29b showed mild correlations at 2 weeks, thus strengthening the role of miRNAs in tooth movement [50].

Hence, the current review provides insight into the exciting domain of epigenetic regulation of microRNA influencing the molecular mechanisms underlying bone and tissue remodeling associated with tooth movement.

### Clinical significance of the study

The current study supports the expression of miRNA present in exosomes existing in GCF of teeth undergoing tooth movement. While change in levels of various mediators like cytokines, enzymes, etc. have been studied earlier in association with varying orthodontic force levels and time intervals, their role is limited to indication of underlying remodeling process consequent to change in mediator expression. On the other hand, miRNA studied in the current review respond to mechanical stimuli and modulate osteoblastogenesis, osteoclastogenesis, and extra-cellular matrix regulation post-transcriptionally at a genetic level. Further, this study has been able to identify the various target genes, e.g., PDCD4 associated with miRNA-21, which can further indicate the pathway of C-Fos regulation directly involved in resorption. Similarly, various other miRNAs studied in tooth movement have been analyzed for identification of mutual target genes for a cumulative effect and further gene ontology performed for HUB gene identification, e.g., COL1A1, COL3A1, COL5A1 etc. which can serve as promising biomarkers for resorption or apposition. It helps in the study of protein-protein interactions which can interpret complex inter-relationships of various processes associated with tooth movement including periodontal inflammation, root resorption or accompanying systemic conditions. This can formulate the basis of target gene therapies for precision or personalized treatment or individualize treatment by upregulation or downregulation of specific genes to bring faster tooth movement with minimal side-effects.

### Limitations of the current study

Although the role of microRNAs is being slowly established in tooth movement, the literature evidence is still scanty and has an unclear risk of bias. The sample size is small with a disproportionate male-female ratio, which may introduce bias in the results of the study. The randomization of subjects or teeth was not performed in the majority of studies; hence the quality of included studies was compromised. The GCF collection protocol was not standardized in all studies as well as oral hygiene regimen and maintenance are not specified. There is no study conducted in saliva in tooth movement, hence only one oral fluid has been studied. The bioinformatics component may not be all-inclusive, as no study to date has been performed in humans to outline all differentially expressed genes in OTM, showing time-dependent variation.

## Conclusions

1. Various microRNAs have been studied in human GCF which may serve as biomarkers for tooth movement, primarily orthodontic canine retraction, including miRNA-21, 27(a/b), 29(a/b/c), 34,146(a/b), 101, and 214. But the literature is scanty with an unclear risk of bias and hence, there is an urgent requirement of outlining a full complement of DEGs to identify the most promising miRNAs as biomarkers for OTM.
2. A statistically significant increase in expression of miRNA-29a/b/c, miRNA-21, miRNA-101 from pre-treatment (prior to initiation of retraction) levels to peak at 4–6 weeks of retraction was seen, but the outcomes are depicted in studies with an unclear ROB. On the other hand, a single study with a low ROB reported miRNA-34a downregulation in negative correlation with MMPs-2,9,14 levels, thus supporting its role in anabolic osteogenesis and apposition.
3. The concentration of miRNAs was found to be higher in the exosome-associated than depleted component of GCF, hence secretory miRNAs can easily be detected in various bio-fluids in exosomes and can further be explored for marker miRNAs and target gene therapy.
4. Bioinformatic analysis revealed 1213 mutual target genes and SMAD4, IGF1, ADAMTS6, COL4A1, COL1A1, COL3A1, FGFR1, COL19A1, FBN1, COL5A1, MGAT4A, LTBP1, MSR1, COL11A1, and COL5A3 as the hub genes. These can be further analyzed for integrated pathways with other associated periodontal and systemic conditions, which might influence tooth movement.
5. Multiple promising miRNA biomarkers were identified: hsa-miR-34a-5p, hsa-miR-29b-2-5p, hsa-miR-29b-3p, hsa-miR-34a-3p, hsa-miR-27a-5p, hsa-miR-29a-5p, hsa-miR-29b-1-5p, hsa-miR-29c-3p, hsa-miR-214-5p, hsa-miR-27a-3p, hsa-miR-29a-3p, hsamiR-146-5p. These can be used in future orthodontic models of tooth movement using different force levels and observation intervals.

### Future directions

1. Differentially expressed gene expression in OTM*:* Full complement of differentially expressed genes (DEGs) in human tooth movement using novel gene testing technologies of RNA sequencing for accuracy in smaller samples. Expression should be monitored in serum, and additionally in oral bio-fluids (GCF and saliva) due to ease in sampling and repeated evaluation.
2. Robust orthodontic study designs: Differences in sex, ethnicity, age (adults and juveniles) need to be addressed to comprehend differences in the bone and tissue remodeling associated with OTM. Larger sample preferred to minimize bias. Different mechanics: orthodontic/orthopedic, functional or accelerated tooth movement require isolation of miRNA markers.
3. Basic research in mechanisms controlling miRNA activity: A detailed analysis for overlapping protein interactions to identify links between various local and systemic conditions including periodontal diseases, neoplasms, etc.
4. Optimal use of miRNA datasets: For computational diagnostic and prediction models and bioinformatic analysis in various orthodontic situations and proposing marker miRNA biosensors for the same.
5. Precision and personalized treatments: Using information of target genes and their pathways by local delivery of miRNA-associated exosomes. This may be beneficial to accelerate treatment time, reduce side effects of root resorption, caries risk, gingival inflammatory conditions, etc.
6. Propose integrated basic research infrastructure in orthodontic specialty: Requirement of trained manpower, adequate bioinformatics training, as well as infrastructure resources for basic research associated to orthodontic departments.

## Supplementary Informations

**Additional file 1.**

## Abbreviations

ALP: Alkaline phosphatase
AST: Aspartate aminotransferase
*Calcr*: Calcitonin receptor
*Cdc42*: Cell division control protein 42
CLP: Cleft lip and palate
DEG: Differential gene expression
ELISA: Enzyme-linked immunoassay
GCF: Gingival crevicular fluid
*Gpr85*: G protein-coupled receptor 85
IL: Interleukin
MAPK: Mitogen-activated protein kinase
miRDB: miRNA database
miRNA: MicroRNA
MMPs: Matrix metalloproteinases
*Nfia*: Nuclear factor I/A
OPG: Osteoprotegrin
OSCC: Oral squamous cell carcinoma
OTM: Orthodontic tooth movement
PCR: Polymerase chain reaction
pg: Picogram
PPI: Protein- protein interaction
PRISMA: Preferred reporting items of systematic reviews and meta-analysis
PLAP-1: Periodontal ligament-associated protein-1
QUADAS: Quality assessment tool for diagnostic accuracy studies
RANKL: Receptor activator of nuclear kappa B ligand
*Srgap2*: SLIT-ROBO Rho GTPase-activating protein 2
TGF-β: Transforming growth factor -β
TNF- α: Tumor necrosis factor- α
TRAP: Tartrate-resistant acid phosphatase
